# SSER: Species specific essential reactions database

**DOI:** 10.1186/s12918-017-0426-0

**Published:** 2017-04-19

**Authors:** Abraham A. Labena, Yuan-Nong Ye, Chuan Dong, Fa-Z Zhang, Feng-Biao Guo

**Affiliations:** 1 Center of Bioinformatics, Key Laboratory for Neuro-Information of Ministry of Education, School of Life Science and Technology, University of Electronic Science and Technology of China, Chengdu, China; 2 Center for Informational Biology, University of Electronic Science and Technology of China, Chengdu, China; 3 College of Computational and Natural Sciences, Dilla University, Dilla, Ethiopia; 4 School of Biology and Engineering, Guizhou Medical University, Guiyang Shi, China; 5 Bioinformatics Center in School of Life Science and Technology, University of Electronic Science and Technology of China, No.4, Section 2, North JianShe Road, Chengdu, 610054 China

**Keywords:** SSER, Database, Essential Reactions, Flux Balance Analysis (FBA), Metabolic Networks

## Abstract

**Background:**

Essential reactions are vital components of cellular networks. They are the foundations of synthetic biology and are potential candidate targets for antimetabolic drug design. Especially if a single reaction is catalyzed by multiple enzymes, then inhibiting the reaction would be a better option than targeting the enzymes or the corresponding enzyme-encoding gene. The existing databases such as BRENDA, BiGG, KEGG, Bio-models, Biosilico, and many others offer useful and comprehensive information on biochemical reactions. But none of these databases especially focus on essential reactions. Therefore, building a centralized repository for this class of reactions would be of great value.

**Description:**

Here, we present a species-specific essential reactions database (**SSER**). The current version comprises essential biochemical and transport reactions of twenty-six organisms which are identified via flux balance analysis (FBA) combined with manual curation on experimentally validated metabolic network models. Quantitative data on the number of essential reactions, number of the essential reactions associated with their respective enzyme-encoding genes and shared essential reactions across organisms are the main contents of the database.

**Conclusion:**

SSER would be a prime source to obtain essential reactions data and related gene and metabolite information and it can significantly facilitate the metabolic network models reconstruction and analysis, and drug target discovery studies. Users can browse, search, compare and download the essential reactions of organisms of their interest through the website http://cefg.uestc.edu.cn/sser.

**Electronic supplementary material:**

The online version of this article (doi:10.1186/s12918-017-0426-0) contains supplementary material, which is available to authorized users.

## Background

Despite their complexity, the reconstructed metabolic networks are important tools to visualize the ‘omics’ data and foster understanding and interpretation of these data in terms of biological functions [1]. Reconstruction of such networks is time intensive and requires extensive effort, costing several months to years depending on the genome size and number of personnel involved [2]. Although the degree of indispensability is not uniformly equal for all of the reactions in the network, each reaction in the metabolic network contributes for the proper functionality of the biological system of the organism in one or other way. Consequently, these reactions are classified as either essential or non-essential. The essential ones are those reactions which are vital for the viability of the organism in a given living conditions than non-essential ones. Some of the reactions are universally essential irrespective of the environment in which the organism is situated, these reactions are identified for a model organism and termed as “super-essential” in the network [3].

Following the whole genome sequencing and biological systems modeling, the number of predictive metabolic network models has been growing significantly. Consequently, tremendous numbers of biological databases storing metabolic pathway information have been developed. Although the efforts have contributed greatly to the understanding of the systems biology of a considerable number of organisms, finding the reaction essentiality data in a centralized repository has given little attention. The existing databases such as KEGG (Kyoto Encyclopedia of Genes and Genomes) [4], BIGG (Biochemical Genetic and Genomic, Systems Biology Research Group of University of California San Diego) [5], Biocyc, Metacyc [6], Ecocyc [7], Bio-models [8], the model SEED [9], GSMN (Genome-Scale Models Database, Tian Jin University) [10], Biosilico [11] and many others offer comprehensive information on biochemical reactions [12], but none of them especially focus on essential reactions. Therefore, building a centralized repository for this class of reactions would be of great value. Essential reactions are potential candidate targets for antimetabolic drug design [3, 13, 14]. Especially if a single reaction is catalyzed by multiple enzymes, then inhibiting the reaction would be a better option than targeting the enzymes itself or the corresponding enzyme-coding gene [15] and this was the key driving force for us towards constructing species-specific essential reactions database (SSER).

The current version (version 1.0) of SSER includes essential biochemical and transport reactions of twenty-six organisms. The reactions were obtained by applying flux balance analysis (FBA) on experimentally validated metabolic network models in in-silico growth conditions in combination with manual curation of each reaction. Besides to storing biochemically essential reactions, SSER can allow the users to obtain information related to the enzyme-coding genes, essential precursors, and products in a defined in in-silico growth conditions. The information from SSER can also have a significant role in biotechnology based industries as essential reactions can be used to increase the yields of production in these industries.

## Construction and content

### Data acquisition and source

Comprehensive, latest and experimentally validated genome-scale metabolic network model versions were downloaded (Nov-Dec 2015) from publically accessible model repositories, mainly BiGG, GSMN and authors’ publications (Additional file 1). It means that a model is selected from multiple versions of an organism, if it is the most up to date, contains comprehensive information and experimentally validated. For instance, we chose to use iJO1366 because it was the most up to date version of *Escherichia coli* K-12 MG1655 at the time of model collection. Furthermore, iJO1366 represents a significant expansion of the E. coli reconstruction than iAF1260 and older versions as it contains greater number of genes, metabolic reactions and unique metabolites [16]. The above criteria were set only to limit the number of models to be considered in the first version of SSER. We put forward to include more models and organisms in future versions. The degree of essentiality of a gene/reaction is crucially dependent on the growth environment, and hence each reaction in our database is supplemented with growth media information. This information was obtained by searching published articles reporting the experimentally validated reconstructions of each organism (see Additional file 2). To investigate the extent of association between essential reactions and essential genes, we downloaded the essential gene information of two organisms selected for the case study, *E. coli* K-12 MG1655 and *Bacillus subtilis* 168 from DEG version 13.0 (Database of Essential Genes) database [17]. We chose the two microbes because they are the most studied and best characterized in terms of their genome annotation, functional characterization, and knowledge of growth behavior [18–20]. See Additional file 3 for the whole workflow.

Recently, computational approaches have become the most powerful techniques over the experimental counterparts in reaction/gene essentiality analysis due to their high sensitivity, speed, accuracy, and low cost [4]. We took an advantage of a constraint-based flux balance analysis (FBA) approach in conjunction with manual curation in constructing SSER. The Constraint-Based Reconstruction and Analysis (COBRA 2.0) [21] toolbox in MATLAB environment was implemented in this regard. FBA is among powerful *in*-*silico* technique which has been widely used in genome-scale metabolic network reconstruction and analysis. A Significant number of studies have also revealed its capability to accurately predict cellular phenotypes from genotypes. For example, in a yeast model, iND750 reconstruction 4,154 *in*-*silico* predicted growth phenotypes across multiple environmental conditions were compared with two large-scale experimental deletion studies showed 83% agreement between the *in*-*silico* and the experimental results [22]. As a second step towards constructing SSER, the models, as downloaded from their source were loaded into MATLAB (MathWorks® R2012b) environment with the Constraint-Based Reconstruction and Analysis (COBRA 2.0) Toolbox [2, 21, 23] and then a single reaction deletion simulation was applied as described in the following section.

### Flux balance analysis (FBA)

Flux balance analysis (FBA) is an approved constraint-based approach which is based on the principle of linear optimization to determine the steady-state reaction flux distribution in a metabolic network by maximizing an objective function [14, 24]. By definition, an essential reaction is a biochemical or transport reaction its deletion abolishes or decreases the cellular growth significantly [25, 26]. The essential reactions in the network models can be determined through single reaction deletion studies. In a single reaction deletion function, a flux value of zero is given to the reaction that is to be removed, or the reaction catalyzed by a particular enzyme is completely removed from the network or switched off. Hence, depending on the value of the Biomass Objective Function (BOF), the fate of each reaction under investigation could be decided [2, 27, 28].

The growth ratio of the mutant to the wild-type denoted as “*grRatio*” in our database and “*Browse*” page of the website, was used to determine essential reactions in each model. Different threshold values of the biomass production rates for gene/reaction essentiality determination has been used, ranging from 0 to 10% growth reduction of the mutant with respect to the wild-type depending on a given substrate conditions and other imposed constraints [26, 29–31]. Yang and coworkers [31] have observed consistency in gene essentiality prediction of the computational method with experimental methods using the biomass production ratio of less than 1% and 0. That is, they obtained consistent results using the cutoffs <1% and 0 separately. They assumed that computationally zero growth can be assessed with biomass production of less than 1e^−6^ for computational noise elimination. In another study, 1% cut-off value was used in determining synthetic reaction lethality analysis [30]. In our work, a reaction is classified as essential if the growth ratio is less than 1% and these reactions were extracted into a separate file for further curation. We thought using this stricter cutoff can reduce the risk of inclusion of the false positives into our collection of essential reactions. A similar threshold value was used in a case study conducted for the validation of single gene deletion function of the COBRA toolbox where the maximum growth rate was defined to be greater than 99% in yeast iDN750 model [21] (see Additional file 4).

Once the reactions that met the above criteria extracted, the next step was to unify the short names (Abbreviations) of the reactions. Searching our database would be troublesome if the reactions were deposited as they were in the models because different researchers follow various methods of nomenclature of biochemical reactions in their reconstructions. Therefore, we looked some way to reorganize and unify the reactions that were identified as essential in each organism. This was achieved by searching in BiGG databases for the abbreviations by using the names of the reactions as a query string. The search results are not always single value but some reaction names are associated with multiple abbreviations. In such conditions, we decide to choose the one with pre-defined reaction parameters such as metabolite type and compartment match with the query reaction. For example, searching BiGG database for the reactions “2 succinyl 6 hydroxy 2 4 cyclohexadiene 1 carboxylate synthase” returned SHCHCS3, SHCHCS2, and 2S6HCCi. Among the results, 2S6HCCi exactly fit our search criteria and hence it is considered as a short name for that particular reaction.

### Database organization

**Fig. 1:**
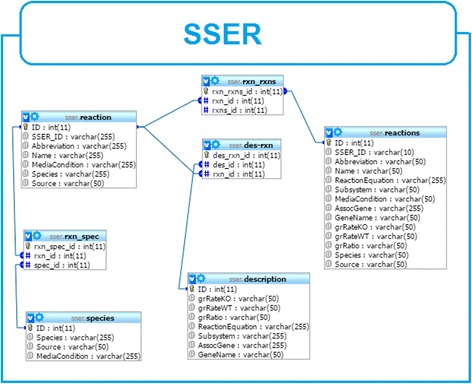
The database schema

The current version (version 1.0) of SSER contains 6077 essential biochemical and transport reactions of twenty-six organisms. It is a relational database built on the top of seven tables, four of which are major contributors whereas the remaining three are bridging tables. The three most important tables include “*reaction*”, “*reactions*”, and “*species*”. The “*reaction*” table lists *SSER*_*ID*, *reaction abbreviation*, *reaction name* and reference’s PubMed ID (*PMID*) for each reaction. The *reactions* table lists the details of each reaction. These include growth rate of knockout strain (*grRateKO*), growth rate wild type (*grRateWT*), growth ratio (*grRatio*), *cutoff*, *reaction equation*, the *subsystem*, *media condition*, *associated gene* and *gene name* if exists. The third table describes the species name and source of the data (see Fig. 1).

## Utility and discussion

**Fig. 2:**
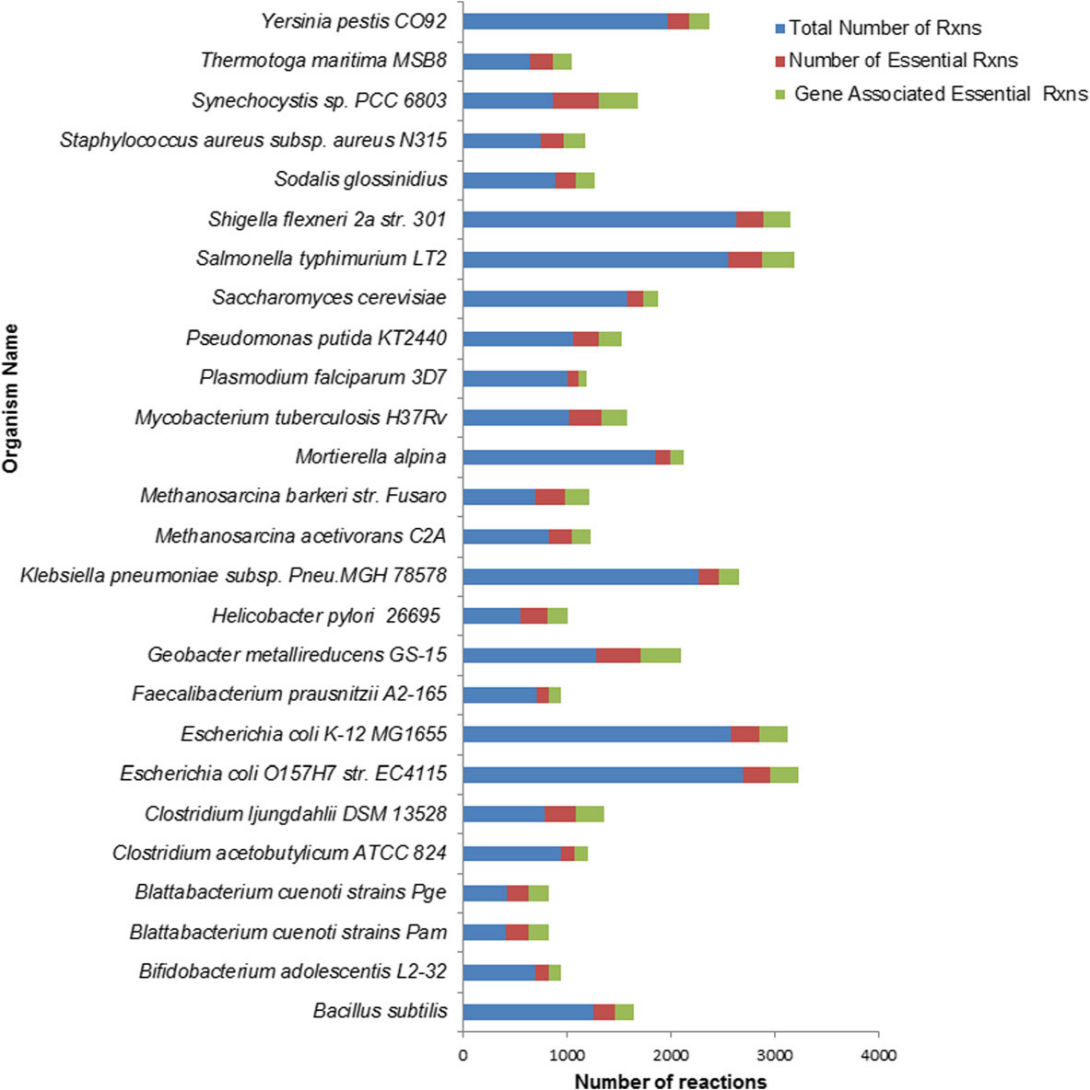
Graph of the total number of reactions, number of essential reactions and number of gene-associated essential reactions

SSER was established with the primary objective of delivering three vital functions to its users. The first and the most important one is to serve as a repository for quantitative data, names, formulae and stoichiometric equations of essential reactions in comparison to the total number of the reactions used in the reconstructions of each organism. Furthermore, a quantitative data about the number of the essential reactions associated with their respective enzyme-encoding genes can also be retrieved from SSER (see Fig. 2).

**Fig. 3:**
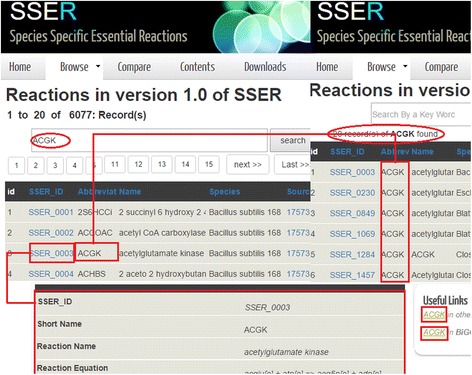
Screen shot of the website. For example, ACGK was searched in the database and 20 results were retrieved indicating that this reaction is essential in 20 species in the database. Clicking on the link at SSER_ID field returns details about each reaction

Users can search for essential reactions of the organisms of their interest in the “*Browse*” page of the website by using a keyword. In addition, the details of each reaction can be browsed by following the link on *SSER*_*ID* field of each reaction. The link returns *reaction equation*, *functional assignment* (*subsystem*), *growth media*, *growth rate knock out* (*KO*), *growth rate wild*-*type* (*WT*), *growth ratio*, *cutoff*, the *SSER*_*ID* and names of the *associated gene*(*s*) of each reaction (see Fig. 3).

The “*Contents*” page is about the statistics of the database and is depicted in the form of tables and graphs. A table of the total number of the essential reactions and number of essential reactions associated with their corresponding enzyme coding genes as well as two graphs of shared essential reactions across the species and essential reaction-essential gene association graphs are included in this page. All the supporting data files such as the models used in this study, in SBML format and all essential reactions comprised in SSER can be downloaded at the “*Download*” page. The “*Download*” page also contains information for programmatic (API) access of SSER. “*Help*” page provides useful information on how to use the database and also it included the description of the headings of the table columns of the browse page. All the references reviewed for each organism are available at “*References*” page.

Secondly, to investigate whether essential reactions are evolutionary conserved or not, we identified the number of essential reactions shared across organisms in our database. To facilitate this task we developed a comparison function which is available on the ‘*Compare*’ page of the website. Users can compare the essential reactions across organisms with similar growth media conditions. As reaction essentiality is mainly determined by environmental condition, the comparison function is particularly limited to the prokaryotes which have grown in glucose minimal medium. Selecting two or more organism from the list on the page and clicking “*Run*” button at the bottom of the page provides a list of the short names and details of the reactions which were isolated as essential in the selected organisms. The result could be opened in the browser and can be downloaded in “.*txt*” file format. For instance, comparing *E. coli* K-12 MG1655 and *Shigella flexneri* 2a strain 301 returned 219 shared essential reactions. This represents 82.3% and 83.2% essential reactions in both organisms, respectively (see Additional file 5). We validated this result against sequence similarity alignment result in genome sequence report of *Shigella flexneri* 2a in which it shared 84.8% (3.9 Mb/4.6 Mb) of its genome with *E. coli* K-12 MG1655 and *Escherichia coli O157* [32]. A study conducted on the evolution of the metabolic network of *E.coli* [33] has also revealed similar result, showing that six *E.coli* strains compared have shared 285 essential reactions in their genomes.

A large number of essential reactions could be shared, particularly if the organisms are closely related on the tree of life. To this end, we calculated the evolutionary distance across 22 prokaryotes using composition vector method [19] and correlated this data with the number of shared essential reactions. For instance, using the same organism as above, *E. coli* K-12 MG1655 and *Shigella flexneri 2a str. 30*, we obtained the shortest calculated Composition Vector Distance (*CVD*) for these organisms (*CVD* = 0.165165606804). But *E. coli* K-12 MG1655 has shared only 124 essential reactions with *Yersinia pestis CO92* which is distantly related to it than *Shigella flexneri* 2a str. 301 (*CVD* = 0.500301106751) (see Additional file 6). Recent studies have also shown that phylogenetically closely related organisms share an evolutionarily conserved core of essential reactions [20, 30, 34, 35]. All the calculated CVD values can be accessed on the “Compare” page of our website.

**Fig. 4:**
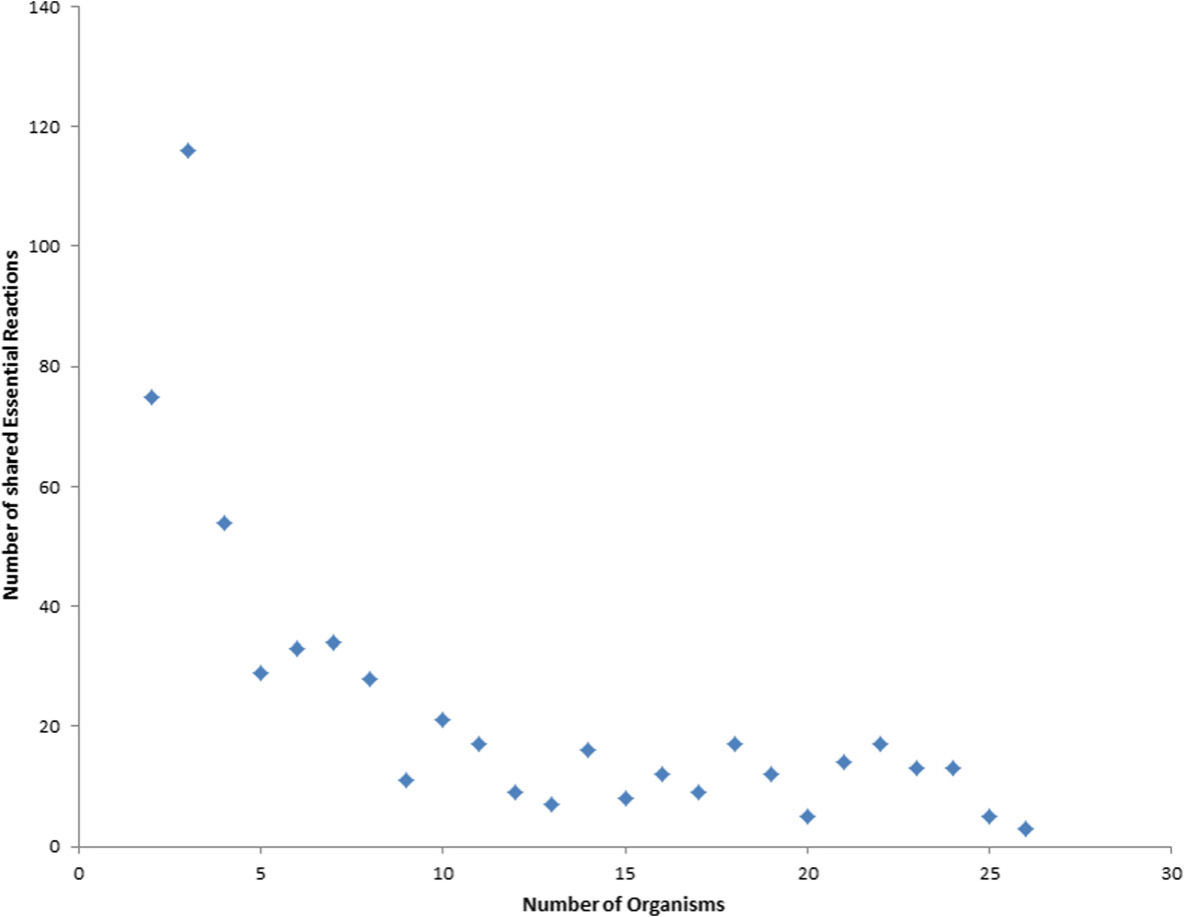
A plot of shared essential reactions across the organisms. The data points represent the number of reactions shared (y-axis) across the organisms (x-axis)

Surprisingly, three reactions, namely CHORS (Chorismate synthase), SHKK (Shikimate kinase) and PSCVT (3phosphoshikimate 1carboxyvinyltransferase), were found to be essential in multiple organisms irrespective of the growth media condition and the phylogeny of the organisms. They were found essential in all 22 prokaryotes in our database. Inspired by the case above, we searched our database for organisms in which a given reaction is essential and identified the number of shared essential reaction across the entire organisms. However, the reason behind this trend needs further investigation which is beyond the scope of this article (see Fig. 4 and Additional file 7).

**Fig. 5:**
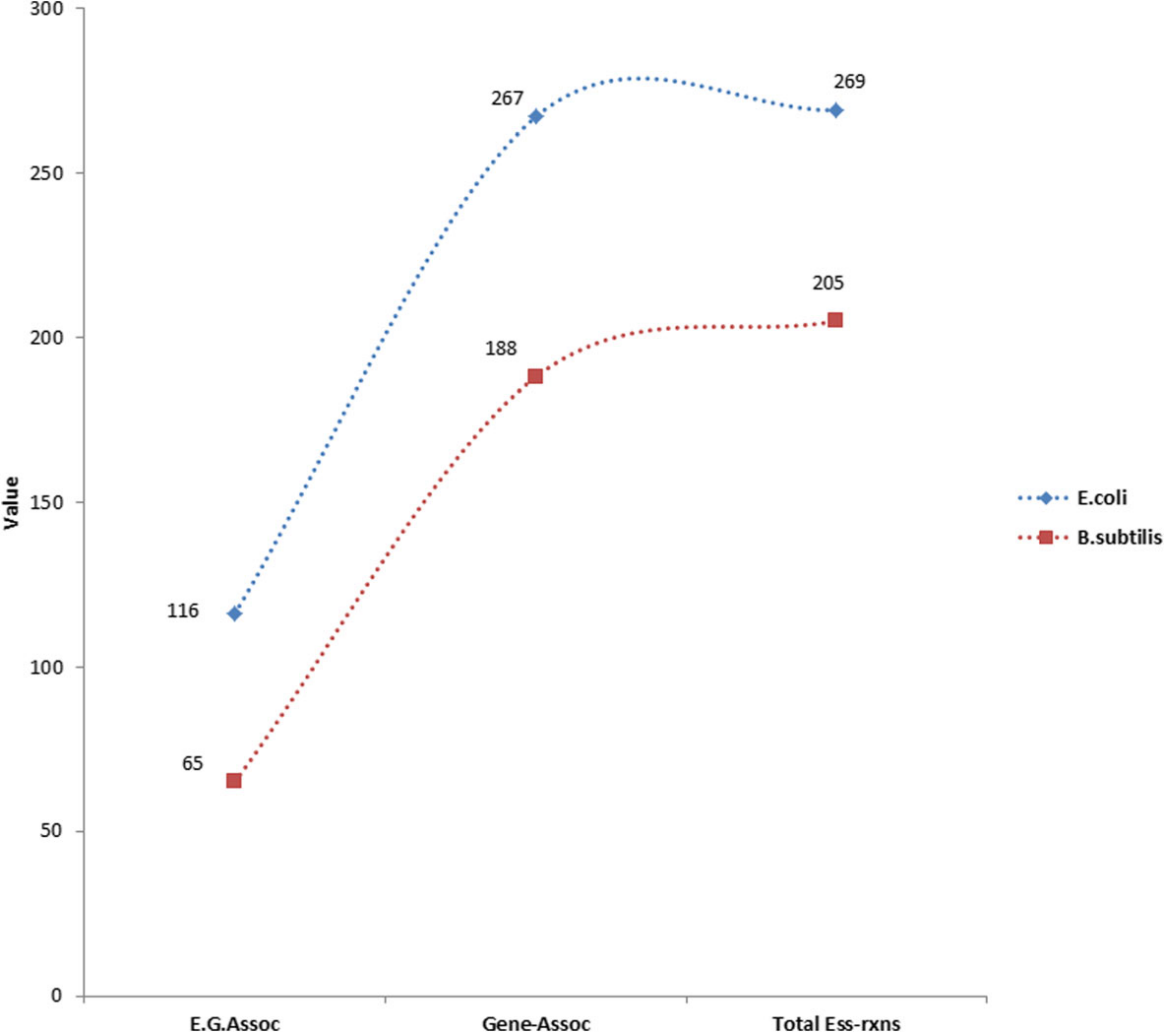
Graphs representing essential reaction-essential gene association data for Escherichia coli K-12 MG1655 and Bacillus subtilis respectively. In the graphs, the *blue* dots represent E.coli and *red* dots represent Bacillus subtilis. Where “Total Ess-rxns” is a total number of essential reactions, “Gene-Assoc” is Gene associated essential reactions and “E.G.Assoc” is Essential Gene associated essential reactions

The third important information in *SSER* is a quantitative data about the number of the essential reactions associated with essential genes. Essential genes have been predicted by removing or switching off enzyme-catalyzed biochemical reactions. If the switching-off of the reaction abolishes or significantly reduces the cellular growth, then the gene that encodes the protein catalyzing that particular reaction is considered to be essential [10, 30, 36]. In this particular case study, *essential reactions*-*essential genes association* analysis of *E.coli* K-12 MG1655 and *Bacillus subtilis 168* strains revealed that 116 and 65 out of 269 and 205 reactions respectively were catalyzed by the enzymes encoded by essential genes. From the case above, we can see that the number of the essential genes is not exactly equal to the number of essential reactions. Therefore, this result alerts us to consider the role of essential reactions in cellular systems studies than solely depending on essential genes information in such studies (see Fig. 5).

## Availability and requirements

SSER is publically accessible via http://cefg.uestc.edu.cn/sser and comprises 6077 essential biochemical reactions of twenty-six species. The website is scripted in HTML5, CSS3, PHP and SQL and tested with Internet Explorer 8, Internet Explorer 7, Firefox, Google Chrome and Safari4.

## Conclusion

The current version of SSER comprises 6077 essential biochemical and transport reactions of twenty-six organisms. The reactions were identified via flux balance analysis (FBA) in conjunction with manual curation on experimentally validated metabolic network models. SSER would be a prime source to obtain essential reactions data and related gene and metabolite information. It can significantly facilitate the metabolic network models reconstruction and analysis, and drug target discovery studies. Furthermore, SSER provides a function for comparing essential reactions across organisms thereby extending its applicability to evolutionary studies. Finally, we put forward to update SSER on a regular basis.

## Additional files

Additional file 1: All models and reactions in SSER. (RAR 3930 kb)

Additional file 2: Growth media information. (XLSX 13 kb)

Additional file 3: Workflow. (TIF 70 kb)

Additional file 4: Names and coefficients of metabolite in BOF. (XLSX 36 kb)

Additional file 5: Shared reactions between E.coli and Shigella flexneri 2a str. 301. (XLSX 23 kb)

Additional file 6: Composition vector distance. (XLSX 14 kb)

Additional file 7: Shared essential reactions across all organisms. (XLSX 870 kb)

## Abbreviations

BOF: Biomass objective function
CBFB: Constraint-based flux balance
COBRA: Constraint based reconstruction and analysis
FBA: Flux balance analysis
GSMN: Genome-scale metabolic models
SBML: Systems biology markup language
SSER: Species specific essential reactions database
