# Supplementary figures and images for: SSER: Species specific essential reactions database
Source: BMC Syst Biol. 2017 Apr 19;11:50. doi: 10.1186/s12918-017-0426-0 (PMC5395902; doi:10.1186/s12918-017-0426-0)

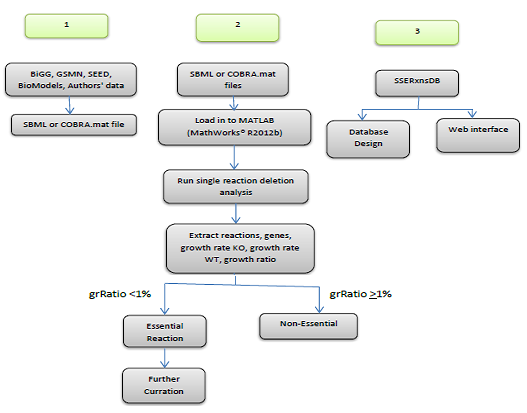

Supplement: Supplementary file 3 — Workflow. (TIF 70 kb) [file 12918_2017_426_MOESM3_ESM.tif]
